# Supplementary material for: Efficacy and safety of next-generation tick transcriptome-derived direct thrombin inhibitors
Source: Nat Commun. 2021 Nov 25;12:6912. doi: 10.1038/s41467-021-27275-8 (PMC8617063; doi:10.1038/s41467-021-27275-8)
Supplement: Supplementary file 1 — Supplementary Information [file 41467_2021_27275_MOESM1_ESM.pdf]

Supplementary Information

Supplementary Fig. 1 to 14: HPLC chromatograms, raw ESI-MS spectra and deconvoluted mass spectra of peptides synthesized for this study.

Supplementary Fig. 15: Thrombin generation curves of variegin, ultravariegin, UFH and bivalirudin in PPP, PRP in the absence of DAPT and PRP in the present of DAPT.

Supplementary Fig. 16: Parameters derived from thrombin generation tests of variegin, ultravariegin, UFH and bivalirudin in PPP, PRP in the absence of DAPT and PRP in the present of DAPT.

Supplementary Fig. 17: Setup of porcine ex vivo stent thrombosis model.

Supplementary Fig. 18: Biolayer-interferometry binding curve fittings for biotinylated ultravariegin against Ab1282 and Ab1283.

Supplementary Fig. 19: Biolayer-interferometry binding curve fittings for biotinylated, scrambled ultravariegin against Ab1282 and Ab1283.

Supplementary Fig. 20: Clotting times of plasma samples drawn from pigs administered with variegin, ultravariegin, UFH and bivalirudin during stent thrombosis model.

Supplementary Fig. 21: Clotting times of plasma samples drawn from pigs administered with variegin, ultravariegin, UFH and bivalirudin, all with a background of DAPT during stent thrombosis model.

Supplementary Table 1: Amino acid sequence, observed and calculated mass of synthesized peptides.

Supplementary Table 2: Slopes of log dose vs log fold change for various parameters in thrombin generation tests.

Supplementary Table 3: Calculated therapeutic indexes of thrombin inhibitors based on half-maximal responses in bleeding and occlusion models.

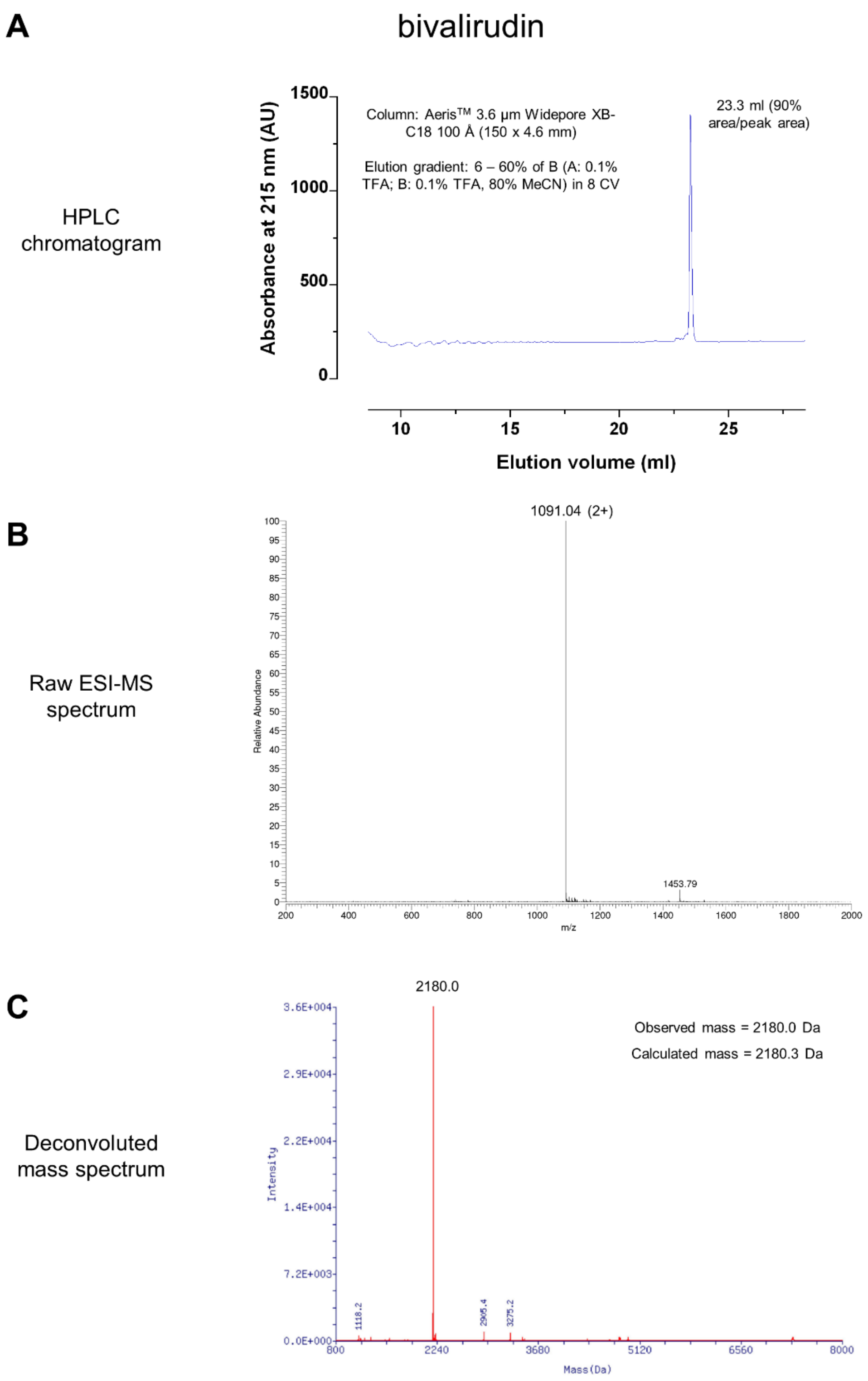

Supplementary Fig. 1. Characterisation of synthetic bivalirudin. (A) RP-HPLC chromatogram, **(B)** Raw ESI-MS spectrum, and **(C)** Deconvoluted mass spectrum of the purified peptide.

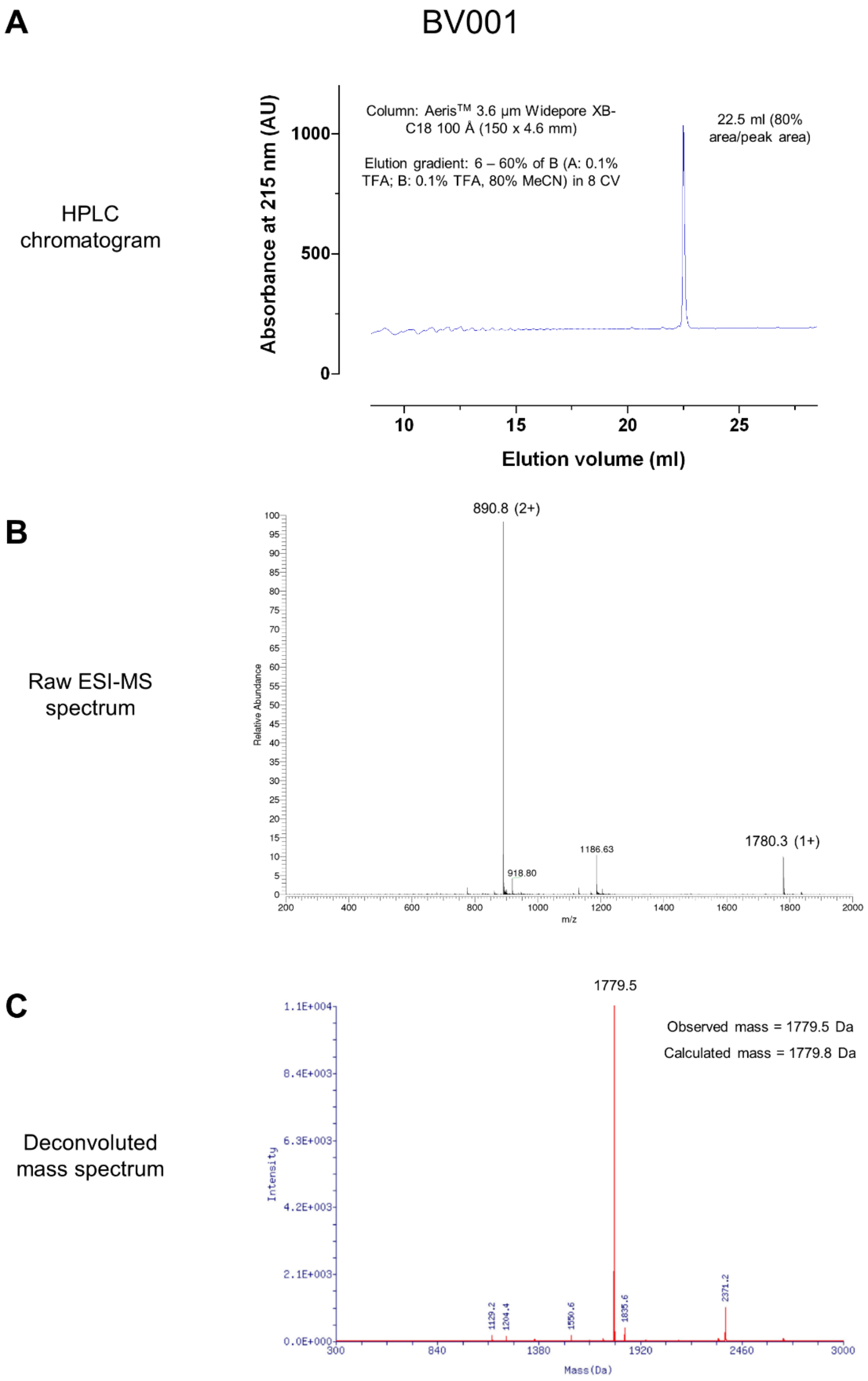

Supplementary Fig. 2. Characterisation of synthetic BV001. (A) RP-HPLC chromatogram, **(B)** Raw ESI-MS spectrum, and **(C)** Deconvoluted mass spectrum of the purified peptide.

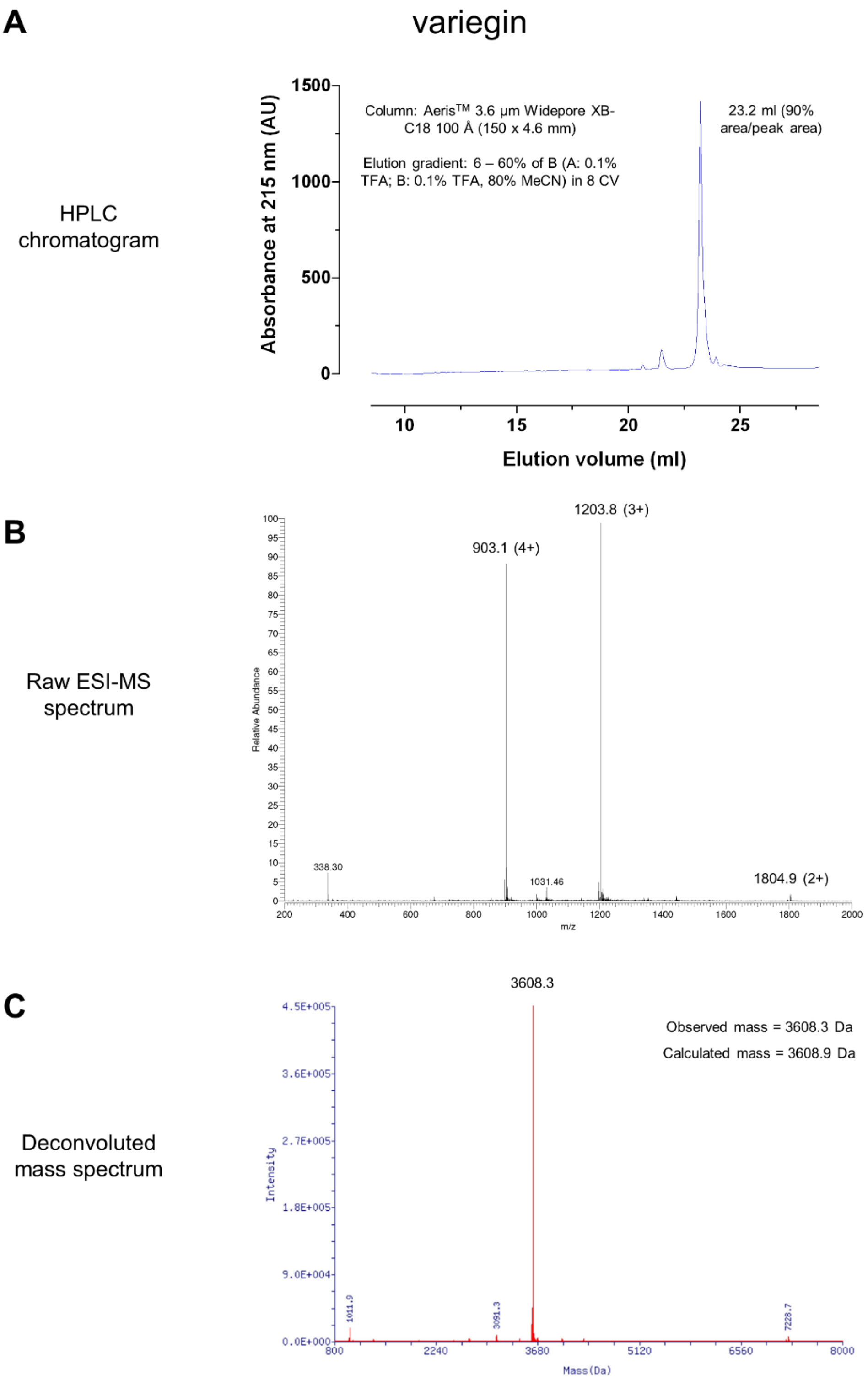

Supplementary Fig. 3. Characterisation of synthetic variegin. (A) RP-HPLC chromatogram, **(B)** Raw ESI-MS spectrum, and **(C)** Deconvoluted mass spectrum of the purified peptide.

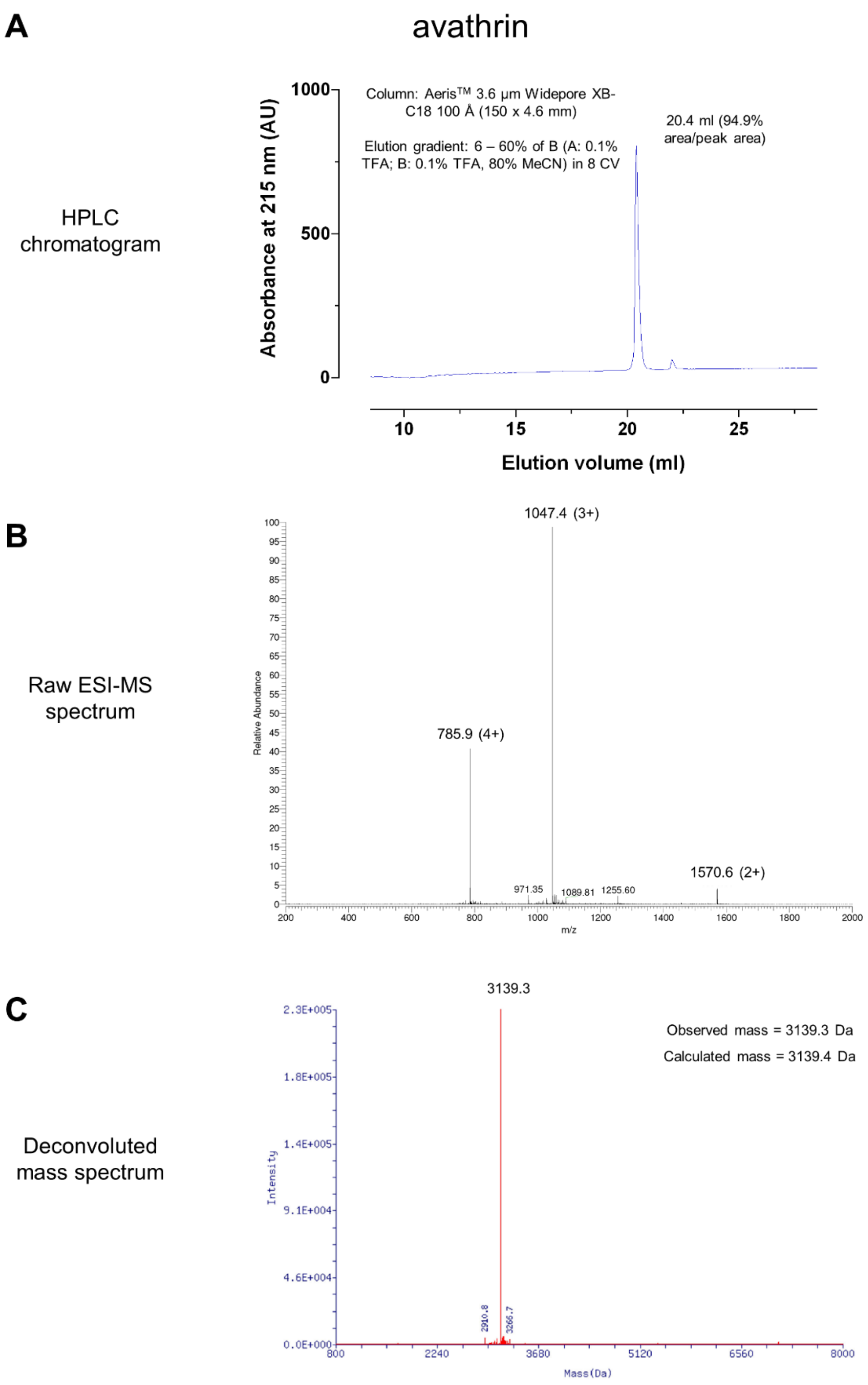

Supplementary Fig. 4. Characterisation of synthetic avathrin. (A) RP-HPLC chromatogram, **(B)** Raw ESI-MS spectrum, and **(C)** Deconvoluted mass spectrum of the purified peptide.

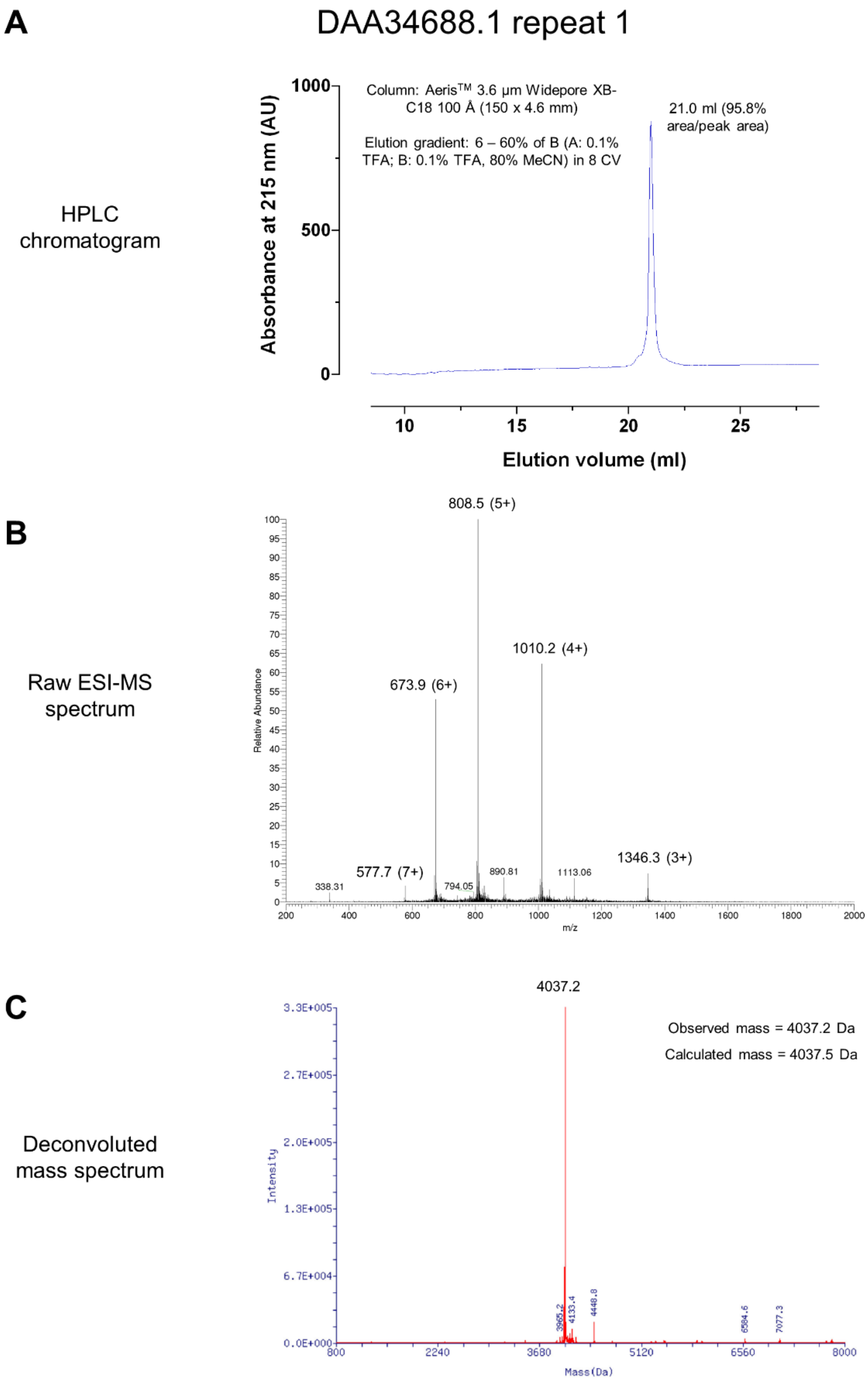

Supplementary Fig. 5. Characterisation of synthetic DAA34688.1 repeat 1. (A) RP-HPLC chromatogram, **(B)** Raw ESI-MS spectrum, and **(C)** Deconvoluted mass spectrum of the purified peptide.

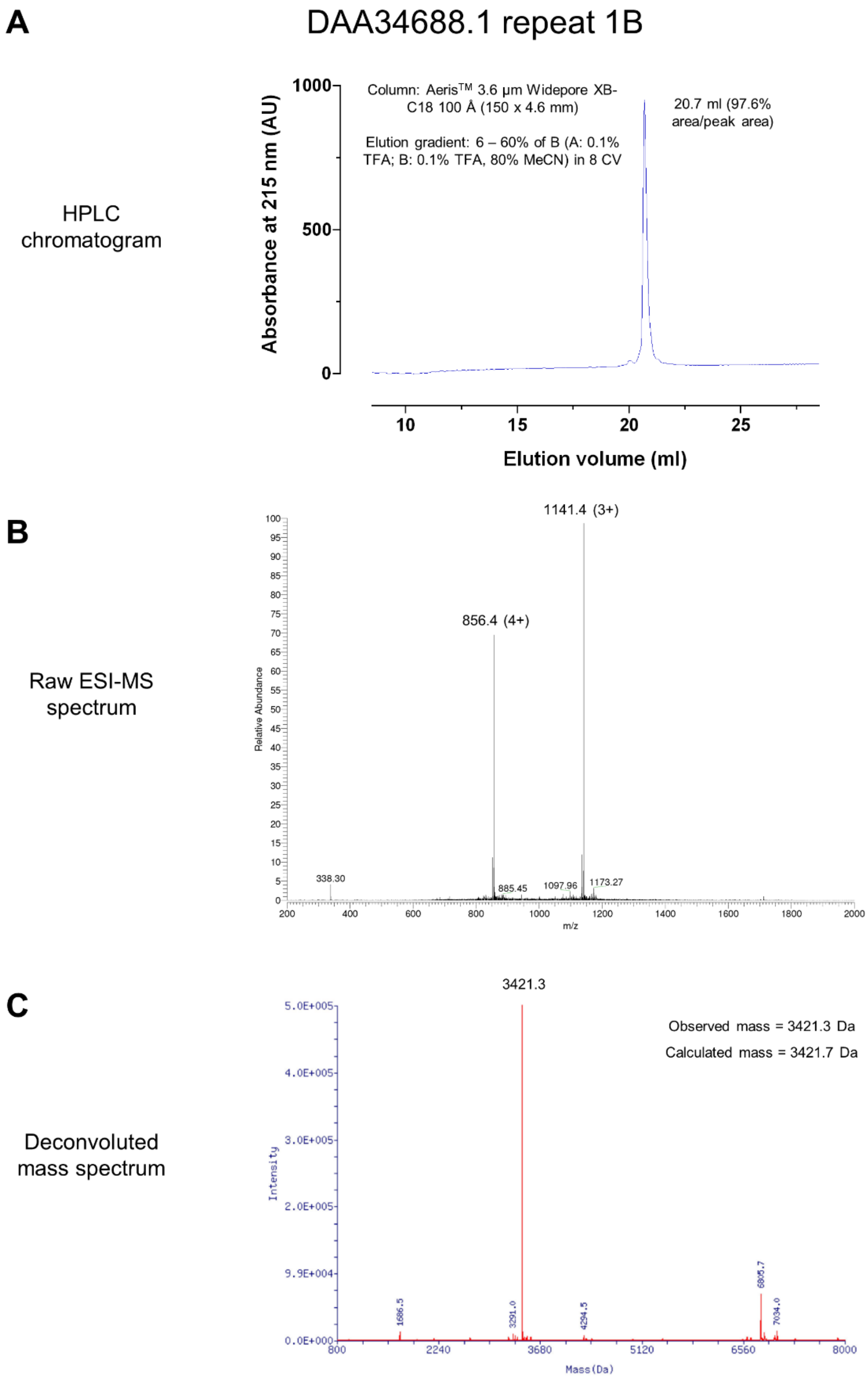

Supplementary Fig. 6. Characterisation of synthetic DAA34688.1 repeat 1B. (A) RP-HPLC chromatogram, **(B)** Raw ESI-MS spectrum, and **(C)** Deconvoluted mass spectrum of the purified peptide.

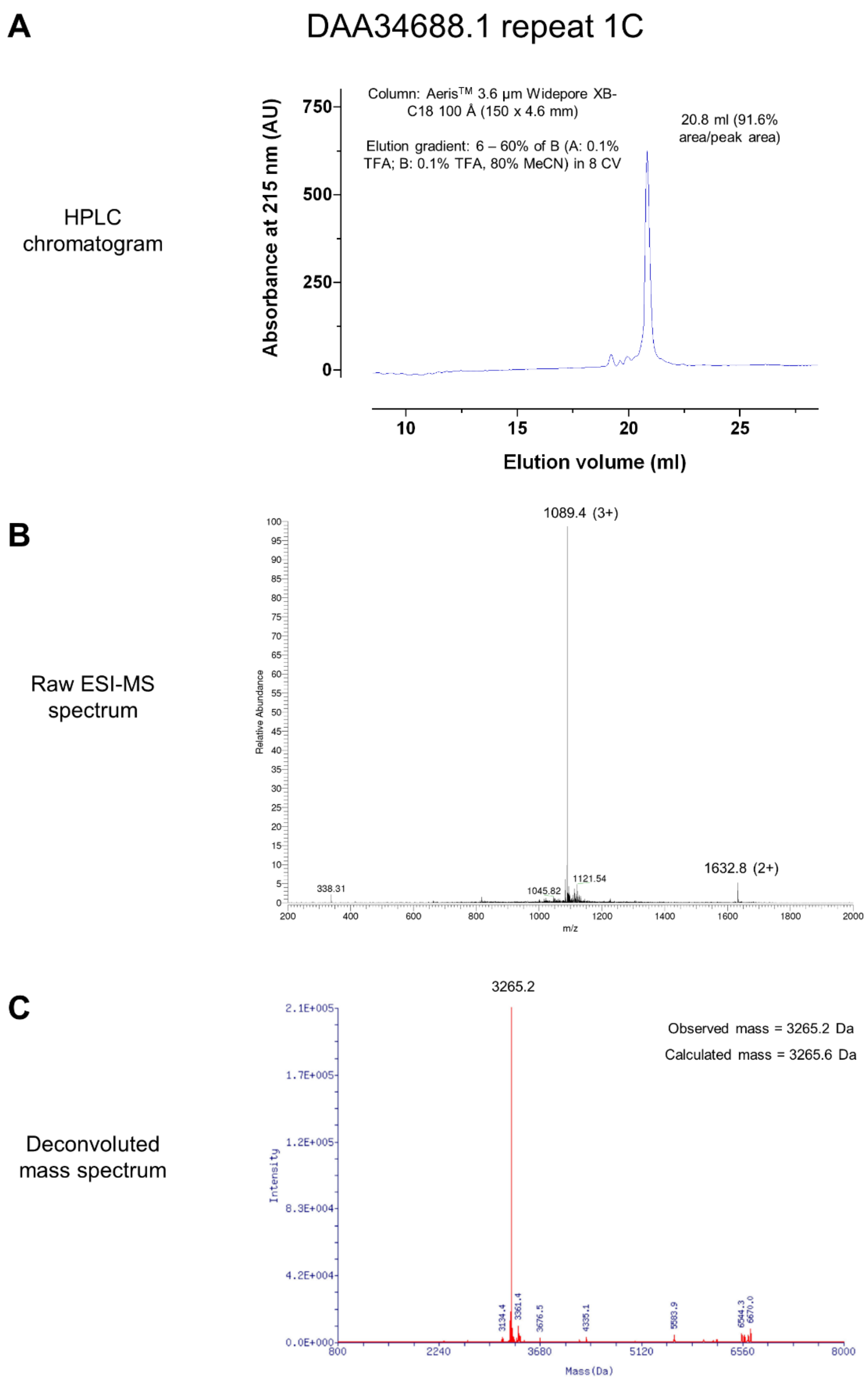

Supplementary Fig. 7. Characterisation of synthetic DAA34688.1 repeat 1C. (A) RP-HPLC chromatogram, **(B)** Raw ESI-MS spectrum, and **(C)** Deconvoluted mass spectrum of the purified peptide.

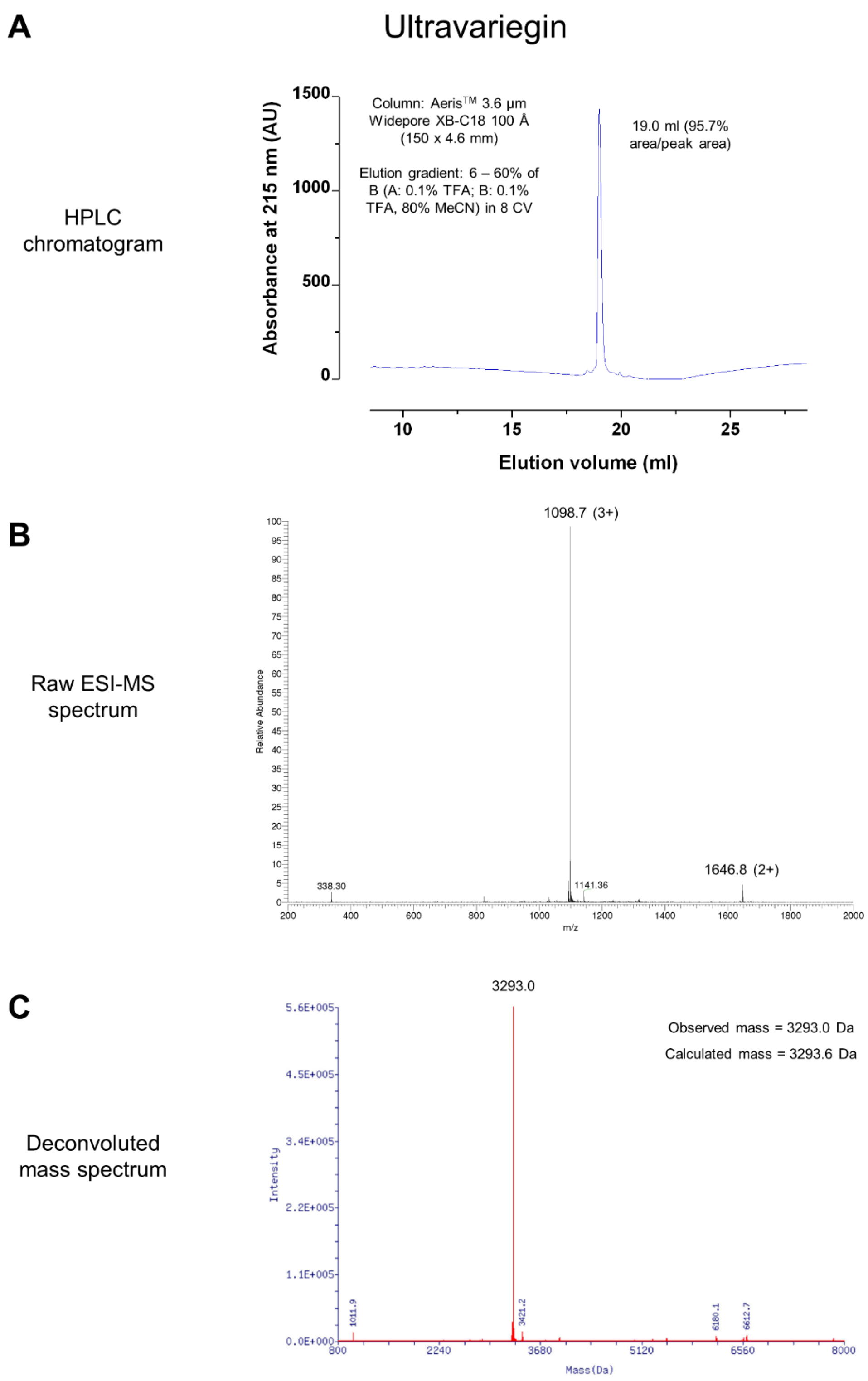

Supplementary Fig. 8. Characterisation of synthetic ultravariegin. (A) RP-HPLC chromatogram, **(B)** Raw ESI-MS spectrum, and **(C)** Deconvoluted mass spectrum of the purified peptide.

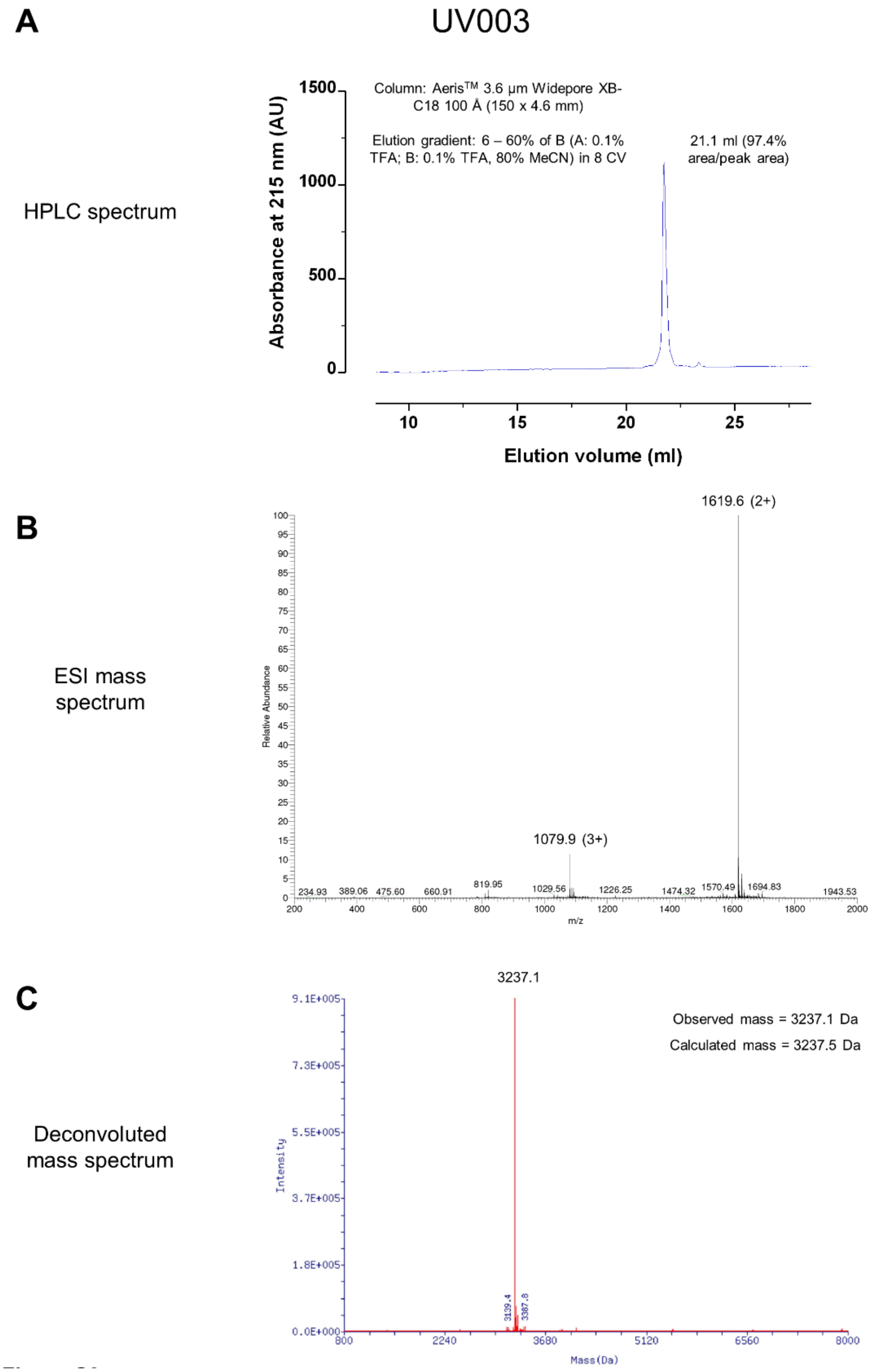

Supplementary Fig. 9. Characterisation of synthetic UV003. (A) RP-HPLC chromatogram, **(B)** Raw ESI-MS spectrum, and **(C)** Deconvoluted mass spectrum of the purified peptide.

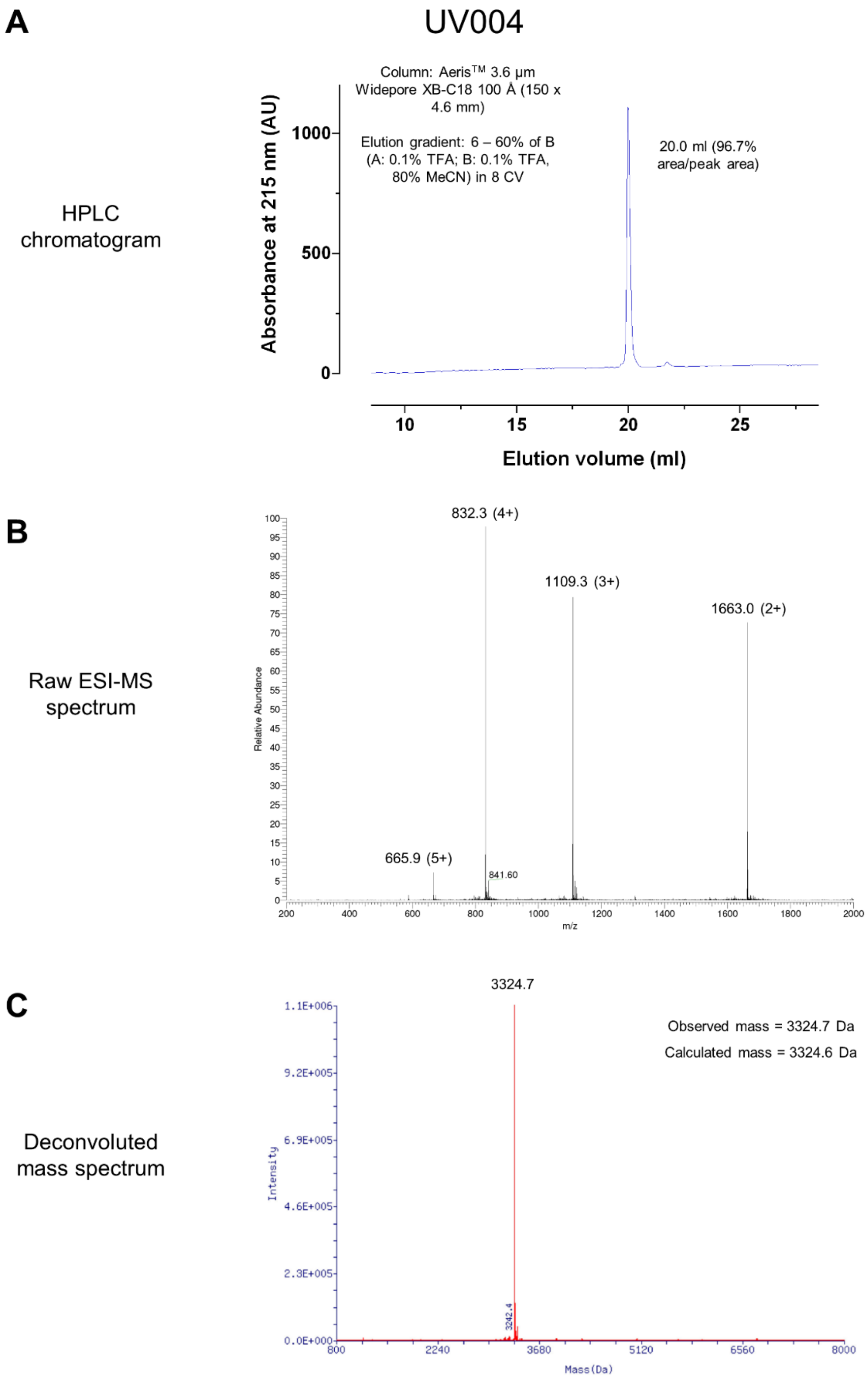

Supplementary Fig. 10. Characterisation of synthetic UV004. (A) RP-HPLC chromatogram, **(B)** Raw ESI-MS spectrum, and **(C)** Deconvoluted mass spectrum of the purified peptide.

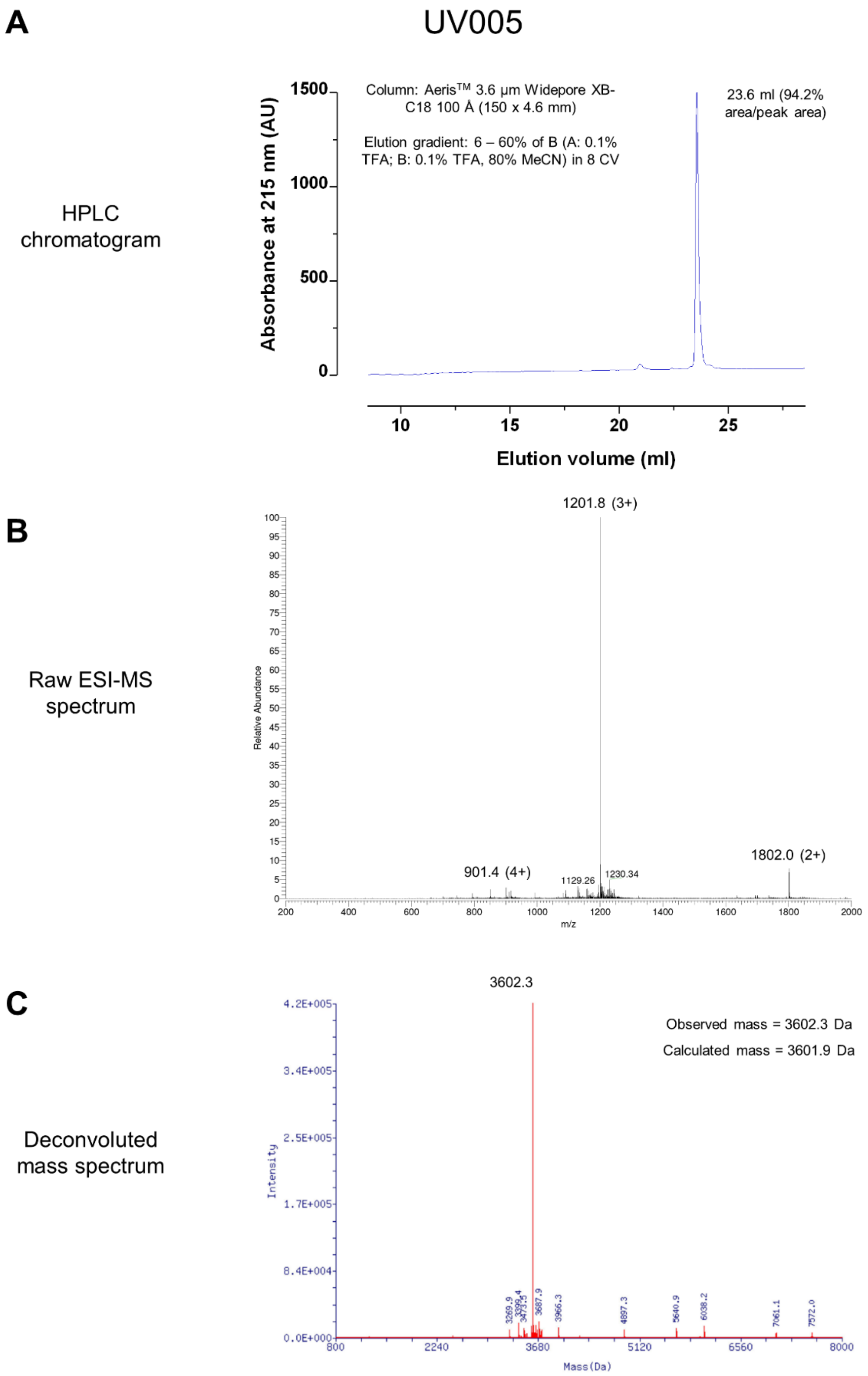

Supplementary Fig. 11. Characterisation of synthetic UV005. (A) RP-HPLC chromatogram, **(B)** Raw ESI-MS spectrum, and **(C)** Deconvoluted mass spectrum of the purified peptide.

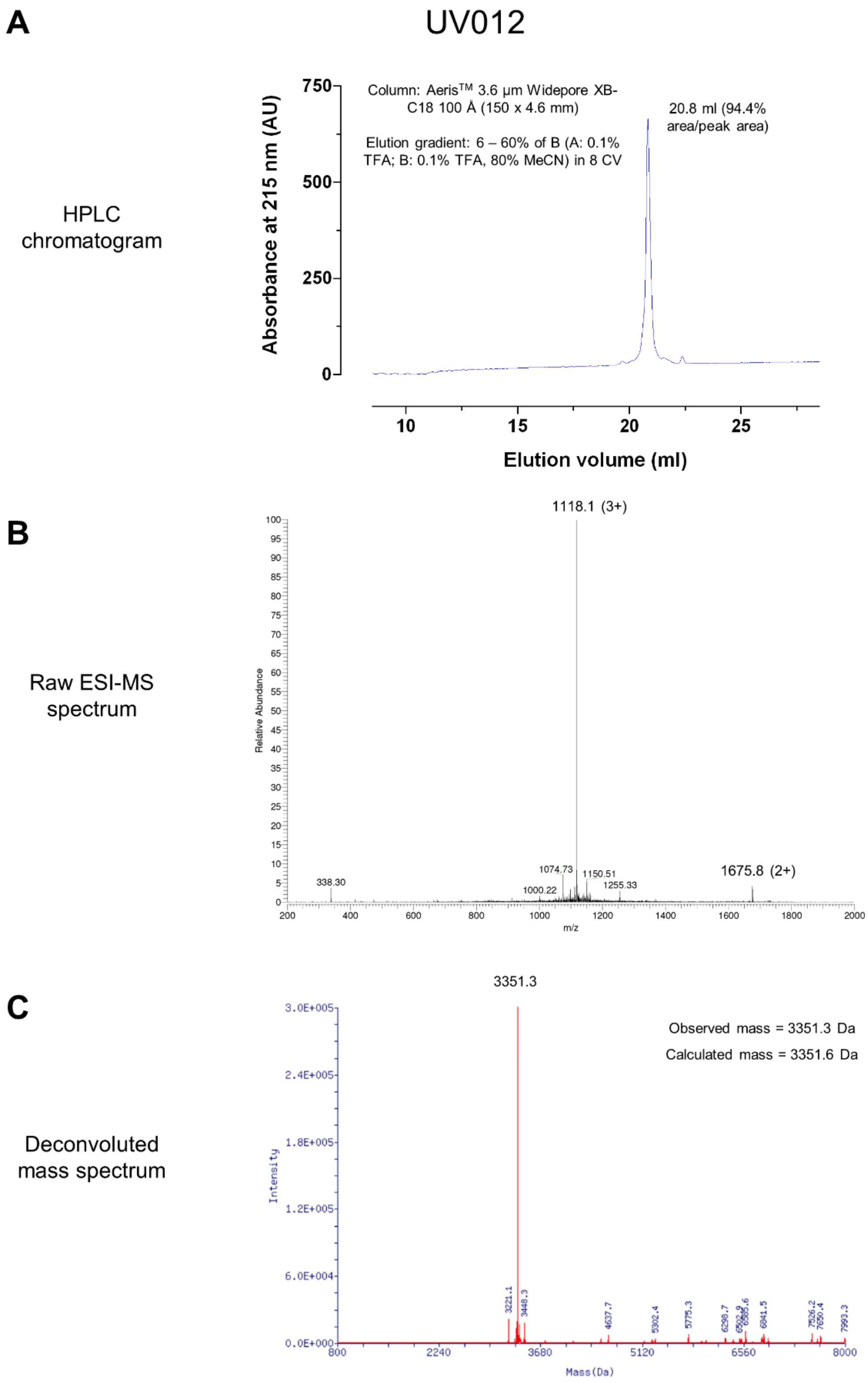

Supplementary Fig. 12. Characterisation of synthetic UV012. (A) RP-HPLC chromatogram, **(B)** Raw ESI-MS spectrum, and **(C)** Deconvoluted mass spectrum of the purified peptide.

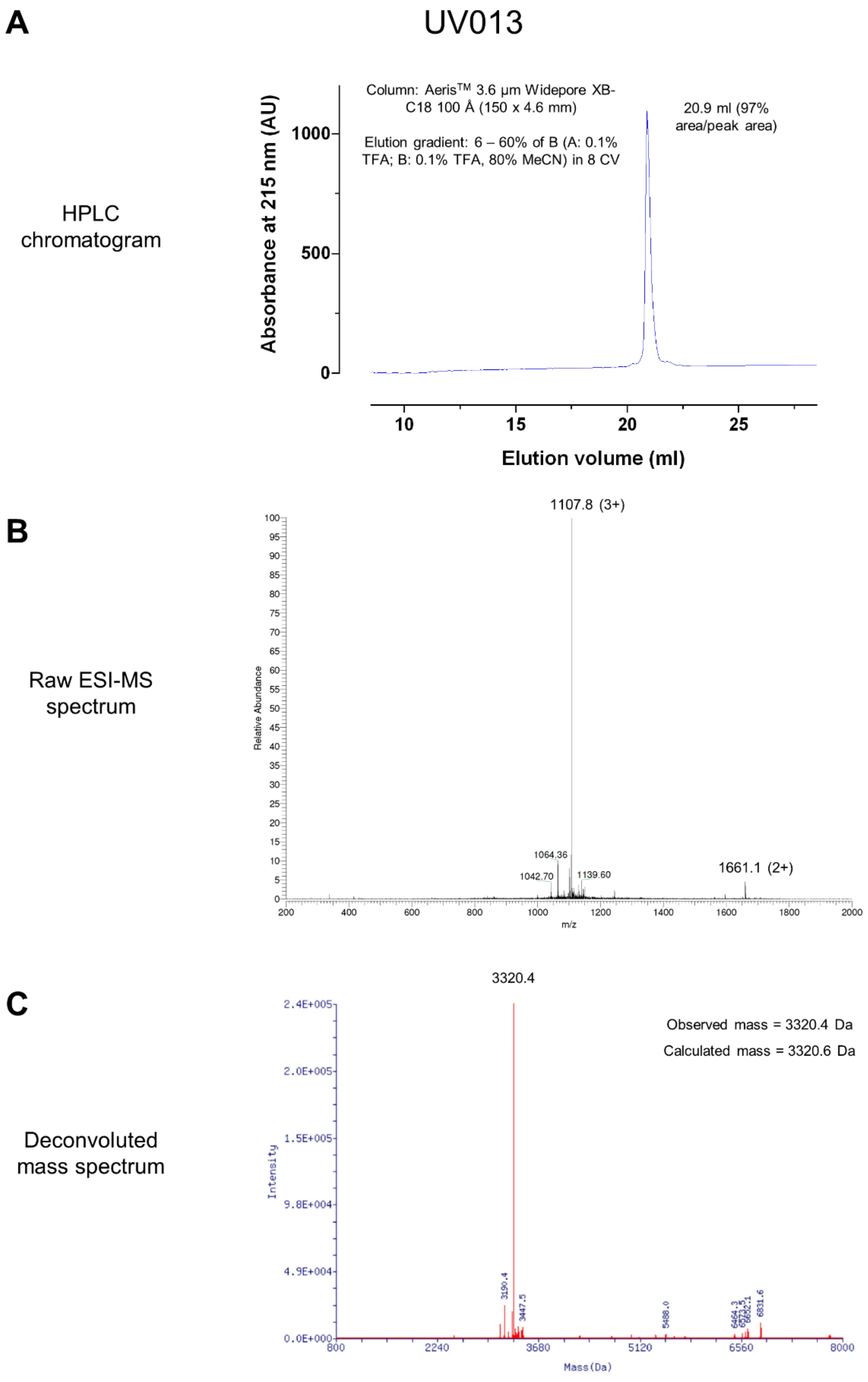

Supplementary Fig. 13. Characterisation of synthetic UV013. (A) RP-HPLC chromatogram, **(B)** Raw ESI-MS spectrum, and **(C)** Deconvoluted mass spectrum of the purified peptide.

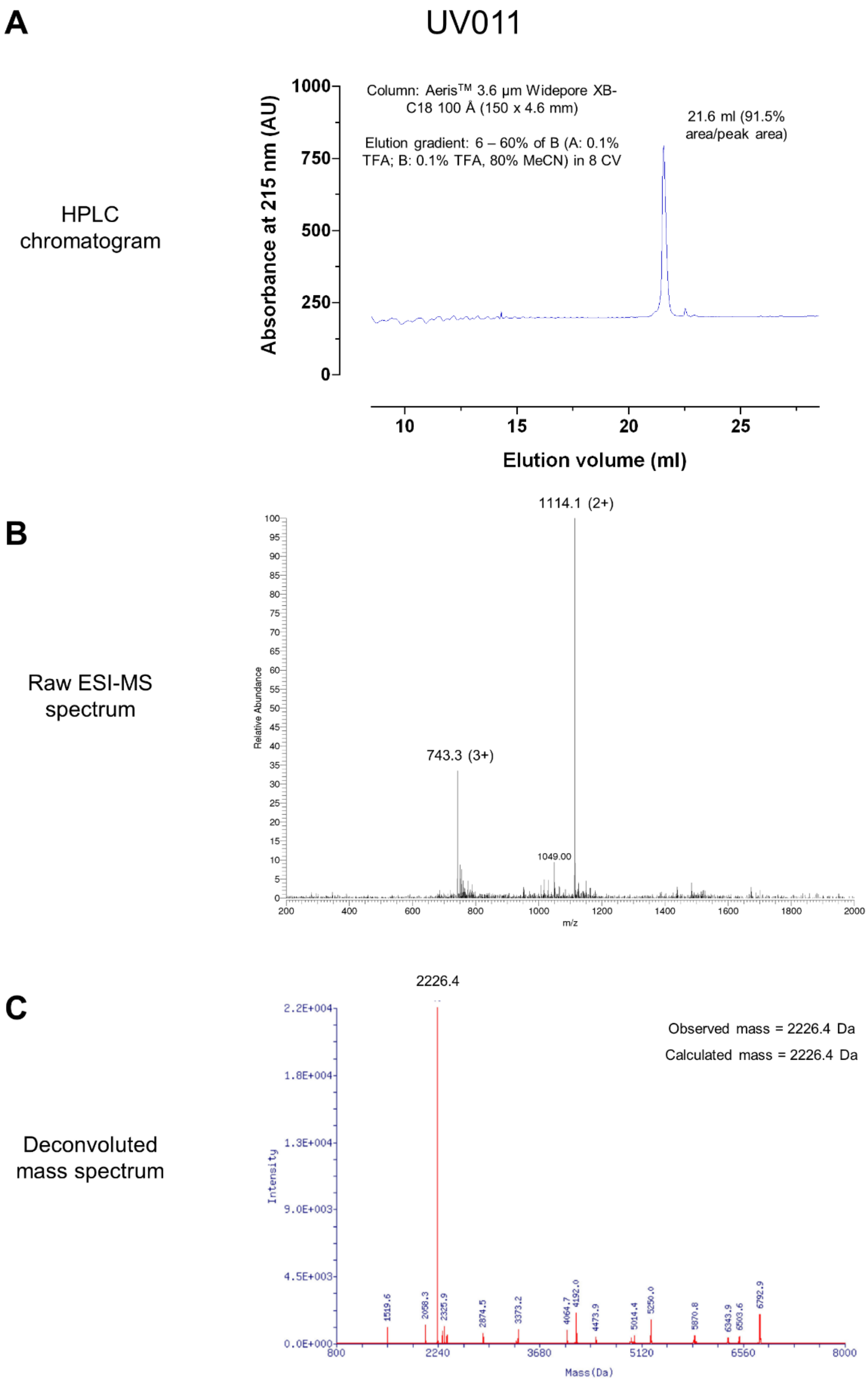

Supplementary Fig. 14. Characterisation of synthetic UV011. (A) RP-HPLC spectrum, **(B)** ESI-MS mass spectrum, and **(C)** Deconvoluted mass spectrum of the purified peptide.

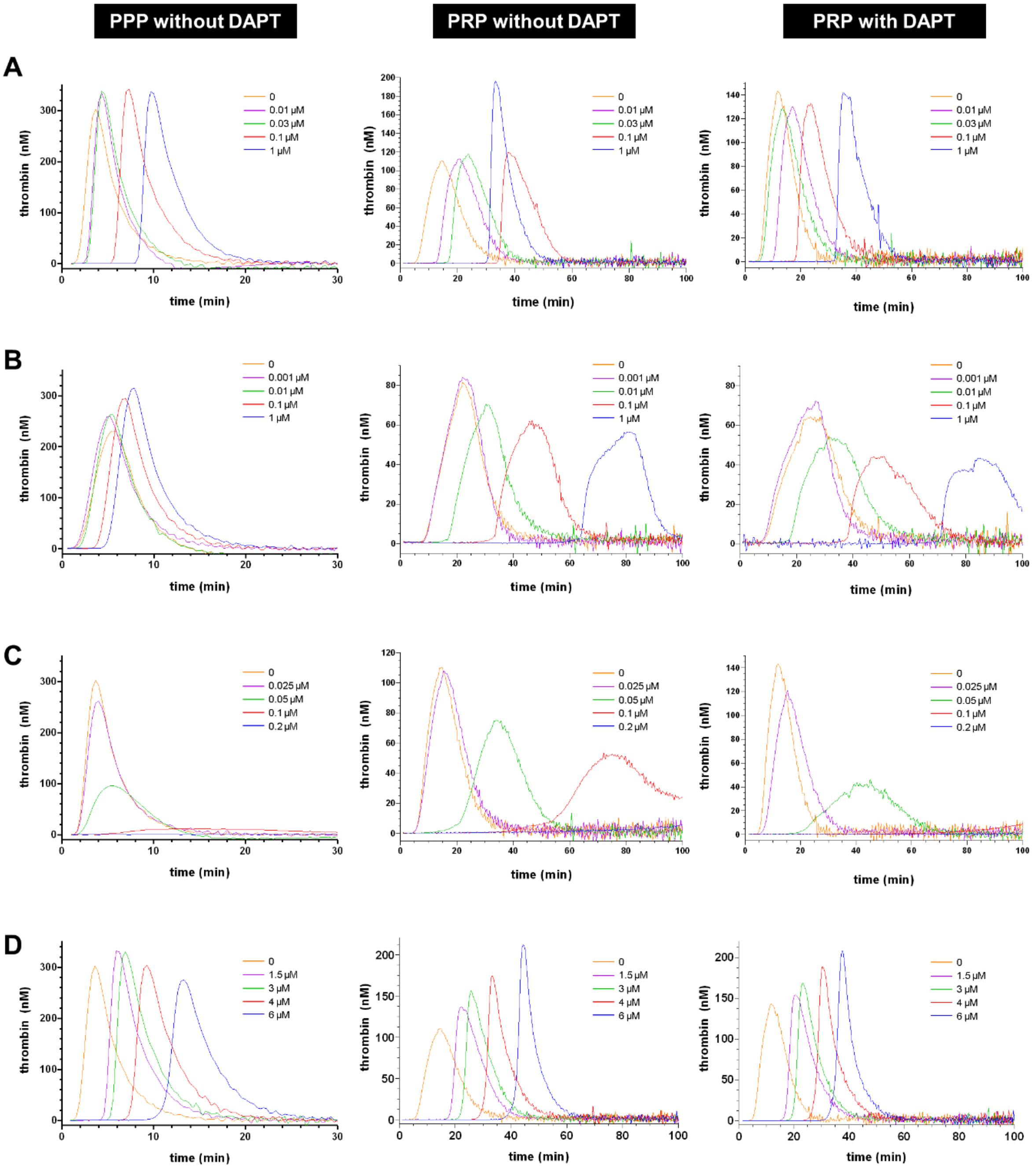

Supplementary Fig. 15. Thrombin generation curves. Representative thrombin generation curves for **(A)** variegin, **(B)** ultravariegin, **(C)** UFH, and **(D)** bivalirudin in PPP, PRP in the absence of DAPT and PRP in the present of DAPT.

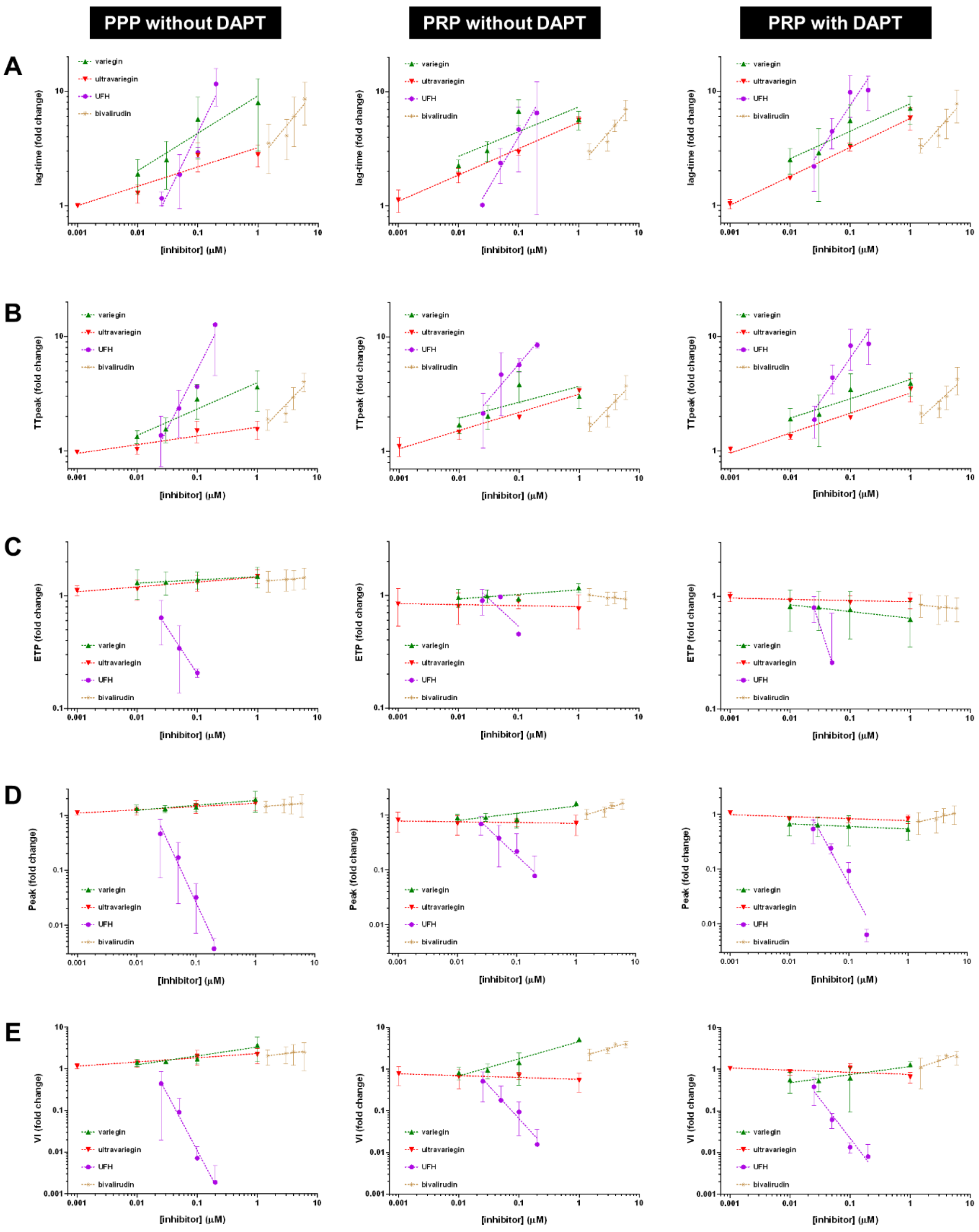

Supplementary Fig. 16. Thrombin generation tests of variegin, ultravariegin, UFH and bivalirudin in PPP, PRP in the absence of DAPT and PRP in the present of DAPT. (A) Lag-time, **(B)** time-to-peak (TTpeak), **(C)** endogenous thrombin potential (ETP), **(D)** peak thrombin concentration (peak), and **(E)** velocity index (VI) are expressed fold change over vehicle controls. Changes in response with increase in concentrations were estimated with linear regression and listed in Supplementary Table 1. Panels A and D were also depicted in the main manuscript and reproduced here for comparisons. Data shown are mean ± SD, *n*=3 biologically independent samples.

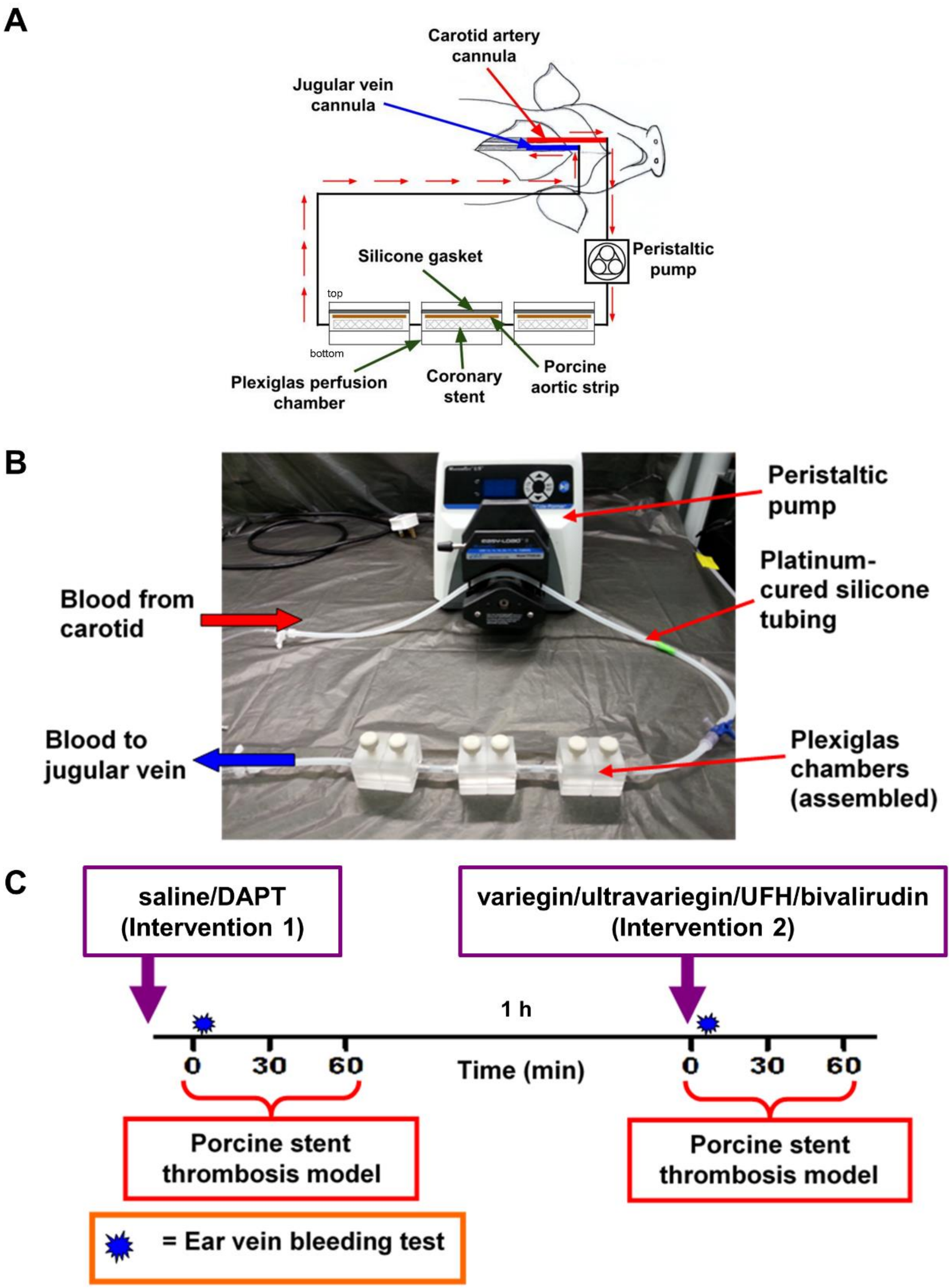

Supplementary Fig. 17. Setup of porcine ex vivo stent thrombosis model. (A) Diagram and photograph **(B)** of the extracorporeal loop used in the porcine stent thrombosis model. **(C)** Timeline showing the administration of drugs, the porcine stent thrombosis model and ear vein bleeding test.

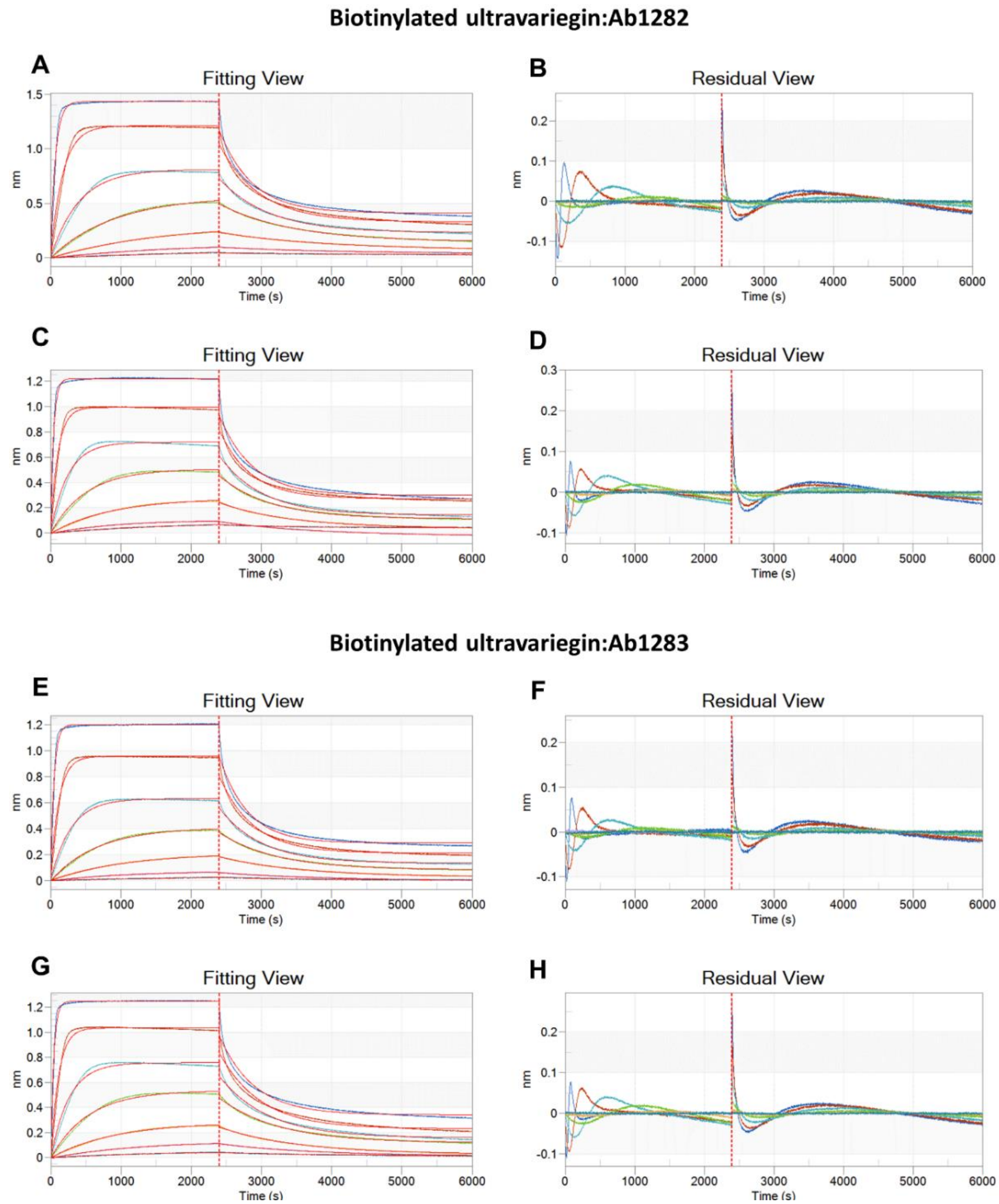

Supplementary Fig. 18. Biolayer-interferometry binding curve fittings for biotinylated ultravariegin against Ab1282 and Ab1283. (A) binding sensorgrams between biotinylated ultravariegin sequence and Ab1282 fitted to a 1:1 kinetic model and **(B)** the residual plot of the fit. **(C)** and **(D)** are from an independent replicate of the experiments. **(E)** binding sensorgrams between biotinylated ultravariegin sequence and Ab1283 fitted to a 1:1 kinetic model and **(F)** the residual plot of the fit. **(G)** and **(H)** are from an independent replicate of the experiments. Concentrations of Ab1282 and Ab1283 used were 20 nM, 6.7 nM, 2.2 nM, 0.74 nM, 0.25 nM, 0.082 nM and 0.027 nM.

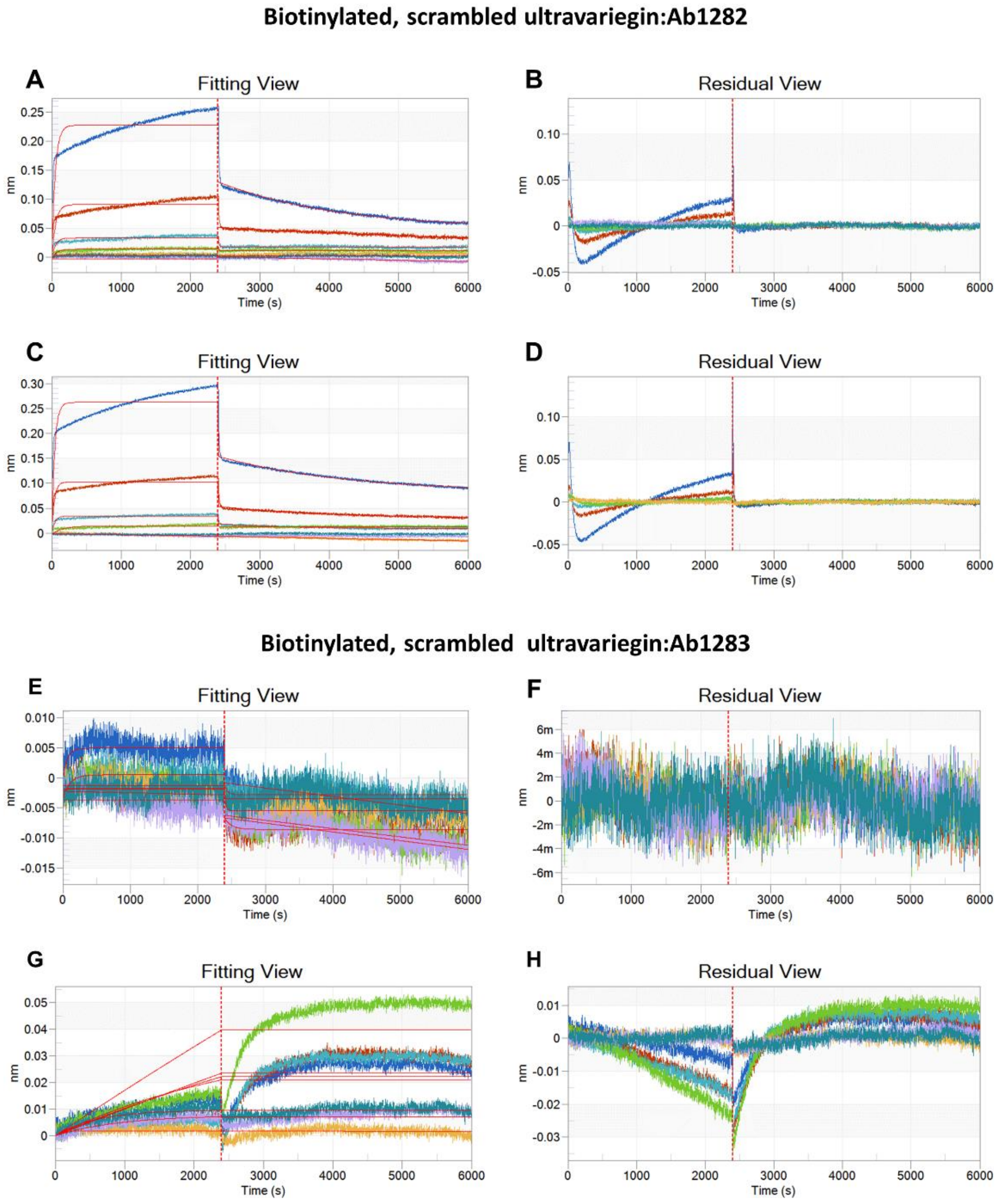

Supplementary Fig. 19. Biolayer-interferometry binding curve fittings for biotinylated, scrambled ultravariegin against Ab1282 and Ab1283. (A) Binding sensorgrams between biotinylated, scrambled ultravariegin sequence and Ab1282 fitted to a 1:1 kinetic model and **(B)** the residual plot of the fit. **(C)** and **(D)** are from an independent replicate of the experiments. **(E)** binding sensorgrams between biotinylated, scrambled ultravariegin sequence and Ab1283 fitted to a 1:1 kinetic model and **(F)** the residual plot of the fit. **(G)** and **(H)** are from an independent replicate of the experiments. No appreciable binding can be detected between biotinylated, scrambled ultravariegin and Ab1283. Concentrations of Ab1282 and Ab1283 used were 20 nM, 6.7 nM, 2.2 nM, 0.74 nM, 0.25 nM, 0.082 nM and 0.027 nM.

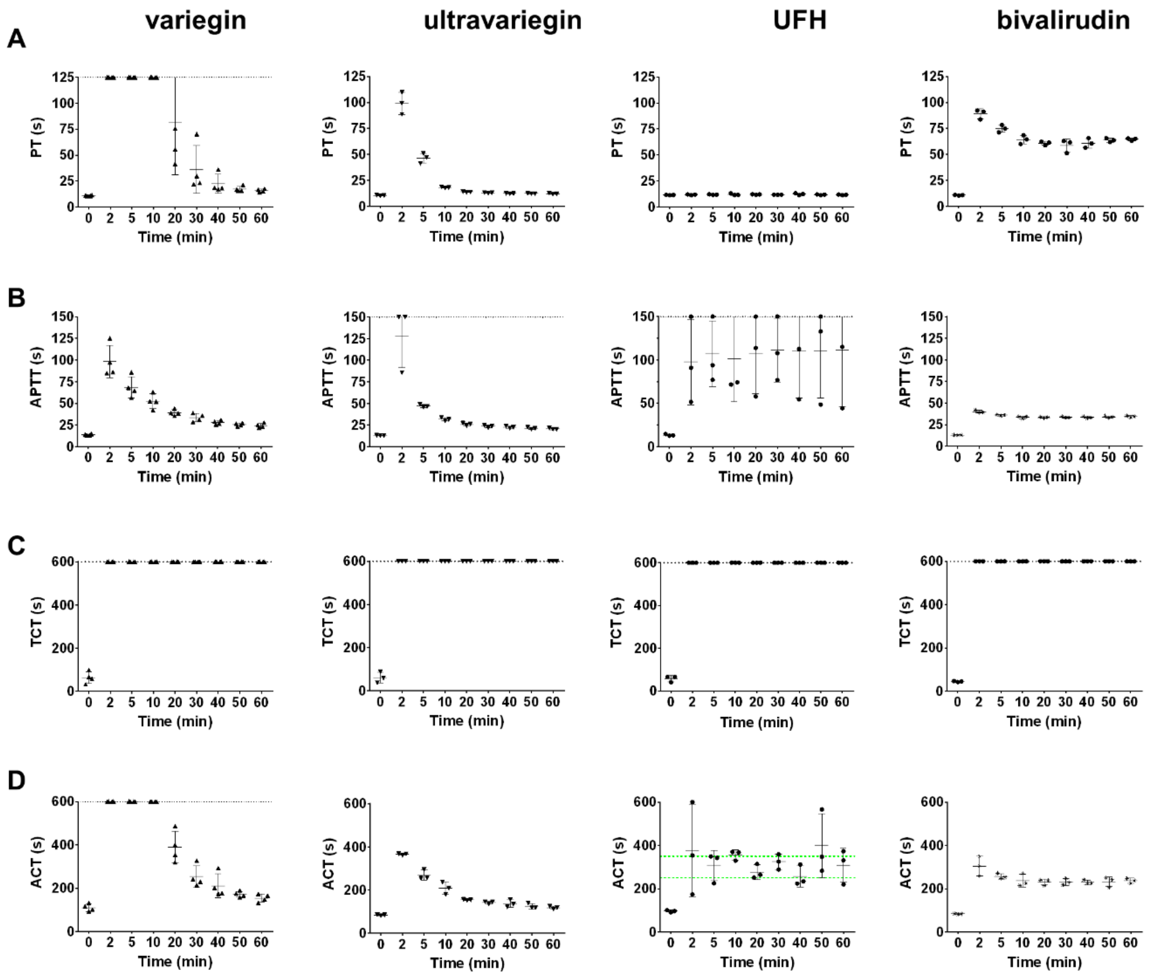

Supplementary Fig. 20. Clotting times of plasma samples drawn from pigs administered with variegin, ultravariegin, UFH and bivalirudin during stent thrombosis model. Samples from representative number of animals were tested. Blood was drawn at different time-points before and after administration of 1 mg/kg variegin (*n*=4), 0.25 mg/kg ultravariegin (*n*=3), 100 U/kg UFH (*n*=3) or 0.75 mg/kg + 1.75 mg/kg/h bivalirudin (*n*=3), respectively. Plasma were prepared and assayed for **(A)** prothrombin time (PT), **(B)** activated partial thromboplastin time (APTT), **(C)** thrombin clotting time (TCT), and whole blood were used for **(D)** activated clotting time (ACT). Assays were stopped if no clots were detected at maximum observation duration, as indicated by black dotted lines. In pigs receiving UFH, ACT was maintained between 250s to 350s (green dotted lines) and top-up of 30 U/kg was administered if ACT falls below 250s during the experiments. Data shown are mean ± SD.

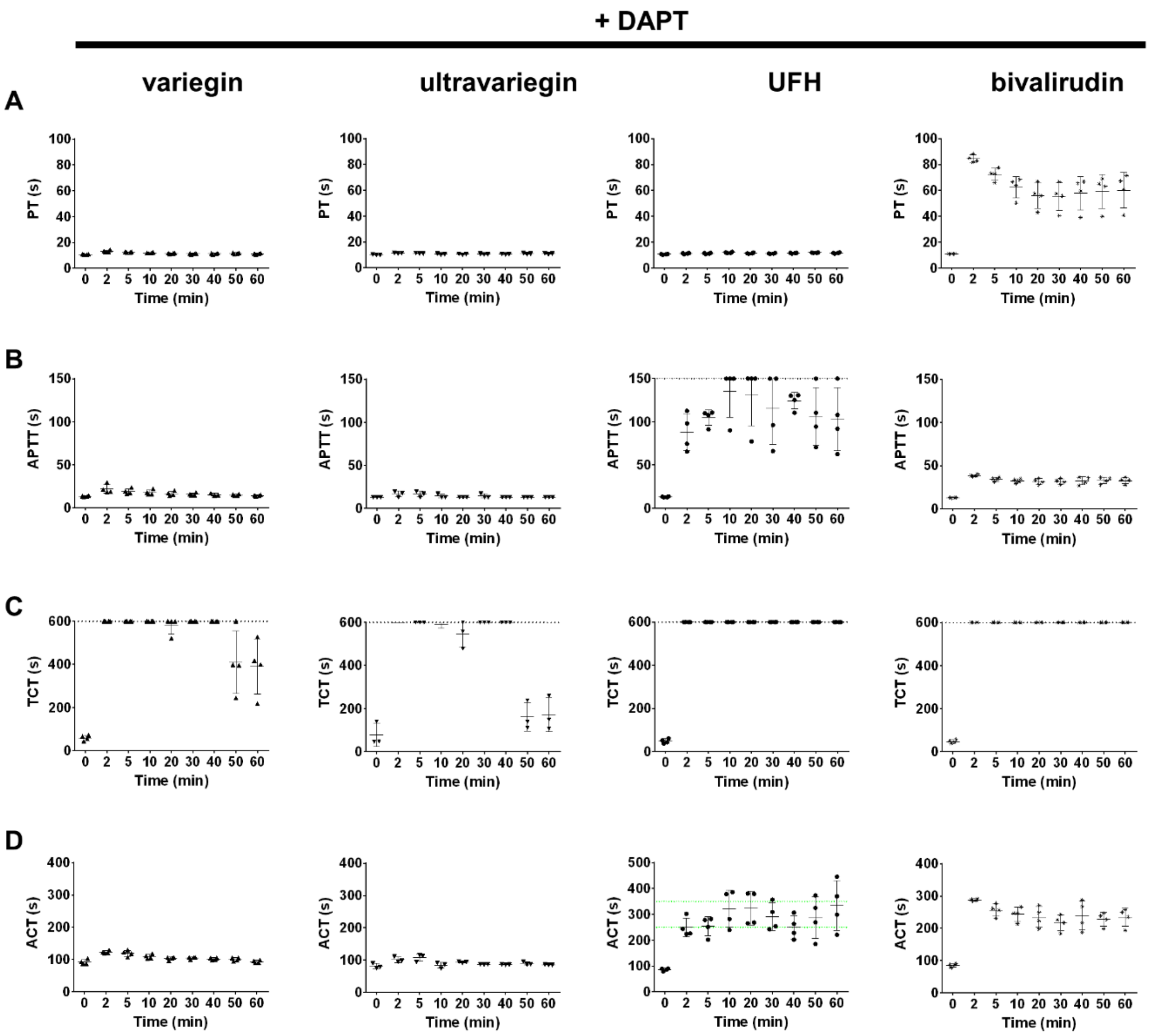

Supplementary Fig. 21. Clotting times of plasma samples drawn from pigs administered with variegin, ultravariegin, UFH and bivalirudin, all with a background of DAPT during stent thrombosis model. Samples from representative number of animals were tested. Blood was drawn at different time-points before and after administration of 0.1 mg/kg variegin (*n*=4), 0.025 mg/kg ultravariegin (*n*=3), 100 U/kg UFH (*n*=4) or 0.75 mg/kg + 1.75 mg/kg/h bivalirudin (*n*=3), respectively. All animals received 300 mg aspirin and 180 mg ticagrelor orally 16 h prior to experiments. Plasma were prepared and assayed for **(A)** prothrombin time (PT), **(B)** activated partial thromboplastin time (APTT), **(C)** thrombin clotting time (TCT), and whole blood were used for **(D)** activated clotting time (ACT). Assays were stopped if no clots were detected at maximum observation duration, as indicated by black dotted lines. In pigs receiving UFH, ACT was maintained between 250s to 350s (green dotted lines) and top-up of 30 U/kg was administered if ACT falls below 250s during the experiments. Data shown are mean ± SD.

Supplementary Table 1. Amino acid sequence, observed and calculated mass of synthesized peptides.

Peptide	Sequence	Observed mass (Da)	Calculated mass (Da)
Bivalirudin	*F*PRPGGGGNGDFEEIPEEYL	2180.0	2180.3
BV001	PGGGGNGDFEEIPEEYL	1779.5	1779.8
Variegin	SDQGDVAEPKMHKTAPPFDFEAIPEEYLDDES	3608.3	3608.9
Avathrin	SGGHQTAVPKISKQGLGGDFEEIPSDEIIE	3139.3	3139.4
DAA34688.1 repeat 1	SDEAVRAIPKMYSTAPPGDFETIPDDAIEEREMKAR	4037.2	4037.5
DAA34688.1 repeat 1B	SDEAVRAIPKMYSTAPPGDFETIPDDAIEER	3421.3	3421.7
DAA34688.1 repeat 1C	SDEAVRAIPKMYSTAPPGDFETIPDDAIEE	3265.2	3265.6
Ultravariegin	SDEAVRAIPKMYSTAPPGDFEEIPDDAIEE	3293.0	3293.6
UV003	SDQGDVAIPKMYSTAPPGDFEEIPDDAIEE	3237.1	3237.5
UV004	SDEAVRAEPKMHKTAPPGDFEEIPDDAIEE	3324.7	3324.6
UV005	SDEAVRAIPKMYSTAPPGDFEEIPEEYLDDES	3602.3	3601.9
UV012	SDEAVRAIPKMYSTAPPGDFEEIPDDEIEE	3351.3	3351.6
UV013	SDEAVRAIPKMYSQAPPGDFEEIPDDAIEE	3320.4	3320.6
UV011	MYSTAPPGDFEEIPDDAIEE	2226.4	2226.4

F in bivalirudin is D-Phenylalanine. See Supplementary Fig. 1 to 14 for raw ESI-MS and deconvoluted mass spectrum of respective peptides.

Supplementary Table 2. Slopes of log dose vs log fold change for various parameters in thrombin generation tests.

		Slope (log dose vs log fold change)			
		**Variegin**	**Ultravariegin**	**UFH**	**Bivalirudin**
**LT**	**PPP**	0.33	0.17	1.1	0.64
	**PRP**	0.22	0.23	0.90	0.61
	**PRP (+ DAPT)**	0.24	0.25	0.79	0.59
**TTpeak**	**PPP**	0.23	0.07	1.0	0.54
	**PRP**	0.14	0.16	0.62	0.54
	**PRP (+ DAPT)**	0.17	0.17	0.76	0.48
**ETP**	**PPP**	0.028	0.043	-0.81	0.043
	**PRP**	0.041	-0.0091	-0.49	-0.060
	**PRP (+ DAPT)**	-0.060	-0.011	-1.6	-0.051
**Peak**	**PPP**	0.088	0.060	-2.3	0.085
	**PRP**	0.13	-0.015	-1.0	0.34
	**PRP (+ DAPT)**	-0.047	-0.036	-2.0	0.25
**VI**	**PPP**	0.21	0.1	-2.7	0.17
	**PRP**	0.41	-0.044	-1.6	0.42
	**PRP (+ DAPT)**	0.19	-0.051	-1.9	0.49

See Supplementary Fig. 16 for respective plots.

Supplementary Table 3. Calculated therapeutic indexes of thrombin inhibitors based on half-maximal responses in bleeding and occlusion models.

	RT_{50} for occlusion	RT_{50} for bleeding	Therapeutic index (TI)
Variegin	1.4 mg/kg	5.3 mg/kg	3.8
Ultravariegin	0.3 mg/kg	2.0 mg/kg	6.7
UFH	225 U/kg	235 U/kg	1.0
Bivalirudin	3.1 mg/kg/h	4.0 mg/kg/h	1.3

See Supplementary Fig. 4 for respective dose-response plots.
