# Supplementary material for: Efficacy and safety of next-generation tick transcriptome-derived direct thrombin inhibitors
Source: Nat Commun. 2021 Nov 25;12:6912. doi: 10.1038/s41467-021-27275-8 (PMC8617063; doi:10.1038/s41467-021-27275-8)
Supplement: Supplementary file 3 — Reporting Summary [file 41467_2021_27275_MOESM3_ESM.pdf]

nature portfolio

Corresponding author(s): Mark Y. Chan

Last updated by author(s): Oct 22, 2021

Reporting Summary

Nature Portfolio wishes to improve the reproducibility of the work that we publish. This form provides structure for consistency and transparency in reporting. For further information on Nature Portfolio policies, see our Editorial Policies and the Editorial Policy Checklist.

Statistics

For all statistical analyses, confirm that the following items are present in the figure legend, table legend, main text, or Methods section.

n/a	Confirmed	
☐	☒	The exact sample size (*n*) for each experimental group/condition, given as a discrete number and unit of measurement
☐	☒	A statement on whether measurements were taken from distinct samples or whether the same sample was measured repeatedly
☐	☒	The statistical test(s) used AND whether they are one- or two-sided<br>*Only common tests should be described solely by name; describe more complex techniques in the Methods section.*
☒	☐	A description of all covariates tested
☒	☐	A description of any assumptions or corrections, such as tests of normality and adjustment for multiple comparisons
☐	☒	A full description of the statistical parameters including central tendency (e.g. means) or other basic estimates (e.g. regression coefficient) AND variation (e.g. standard deviation) or associated estimates of uncertainty (e.g. confidence intervals)
☐	☒	For null hypothesis testing, the test statistic (e.g. *F*, *t*, *r*) with confidence intervals, effect sizes, degrees of freedom and *P* value noted<br>*Give P values as exact values whenever suitable.*
☒	☐	For Bayesian analysis, information on the choice of priors and Markov chain Monte Carlo settings
☒	☐	For hierarchical and complex designs, identification of the appropriate level for tests and full reporting of outcomes
☐	☒	Estimates of effect sizes (e.g. Cohen's *d*, Pearson's *r*), indicating how they were calculated

Our web collection on statistics for biologists contains articles on many of the points above.

Software and code

Policy information about availability of computer code

Data collection	Tecan Magellan 7.0 software was used to collect absorbance reading in Tecan InfinitePro M200 microplate reader. Thermo Xcalibur 2.2 was used to collect ESI-MS data on LCQ Fleet Ion Trap MS. Thrombinoscope 5.0 software was used to collect thrombin generation data. LabChart 7.0 Pro was used to collect Doppler flow probe data.
Data analysis	Prism 6.0 software was used for data and statistical analysis. Mass spectra were deconvoluted using ProMass for Xcalibur 3.0 software. Sysmex CWA analysis software IPU version 18 was used for APTT and CWA analysis. Thrombinoscope 5.0 software was used to analyse thrombin generation curves. FortéBio Data Analysis 9.0 software was used for BLI data analysis.

For manuscripts utilizing custom algorithms or software that are central to the research but not yet described in published literature, software must be made available to editors and reviewers. We strongly encourage code deposition in a community repository (e.g. GitHub). See the Nature Portfolio guidelines for submitting code & software for further information.

Data

Policy information about availability of data

All manuscripts must include a data availability statement. This statement should provide the following information, where applicable:

- Accession codes, unique identifiers, or web links for publicly available datasets
- A description of any restrictions on data availability
- For clinical datasets or third party data, please ensure that the statement adheres to our policy

Transcriptomic data utilised have been previously published34 and not generated in the present paper. The sequence data used to inform the design of ultravariegin as depicted in Fig. 1 is publicly available in UniProt under accession number P85800 (https://www.uniprot.org/uniprot/P85800) and in GenBank under accession number BAD29729.1 (https://www.ncbi.nlm.nih.gov/protein/BAD29729), DAA34688.1 (https://www.ncbi.nlm.nih.gov/protein/DAA34688.1), DAA34258.1

(https://www.ncbi.nlm.nih.gov/protein/DAA34258.1), and DAA34160.1 (https://www.ncbi.nlm.nih.gov/protein/DAA34160.1). Sequences of antibodies tested as reversal agents are available upon reasonable request under cover of a non-disclosure agreement until the application for intellectual property is completed and published. The remaining data generated in this study are provided in the Article, Supplementary Information, or Source Data file. Source data are provided with this paper.

Field-specific reporting

Please select the one below that is the best fit for your research. If you are not sure, read the appropriate sections before making your selection.

☒ Life sciences ☐ Behavioural & social sciences ☐ Ecological, evolutionary & environmental sciences

For a reference copy of the document with all sections, see nature.com/documents/nr-reporting-summary-flat.pdf

Life sciences study design

All studies must disclose on these points even when the disclosure is negative.

Sample size	No statistical method was used to predetermine sample size. All in vitro enzymatic and thrombin generation experiments were repeated at least three times independently. Sample sizes were chosen based on our prior experience and consistent with similar experiments in the literature. In vitro experiments involving reversal agents were performed independently 2 times due to limited availability of expressed antibodies. At least 5-8 rats per group were used for in vivo experiments except 3-4 rats used for two highest doses in ultravariegin occlusion and bleeding experiments where the results are consistently above maximum observation durations. For pig experiments, at least 3-4 pigs per treatment group were used. After we have established that control treatments (saline or DAPT) can be performed on the same pig used subsequently for drug treatments with adequate wash-out period, we are able to perform more experiments for control treatments (saline n=7, DAPT n=12).
Data exclusions	One data point in Fig. 3C & 3D at highest concentration of variegin were excluded as thrombin generation assay returned with an error.
Replication	Independent biological replications of all data were performed, in n numbers as indicated in Methods and Legends. All reported attempts at replication were successful.
Randomization	Treatment and dosage administered to animals are given in a random order. Blood/plasma from three healthy volunteers were collected and each samples received the same treatments and analysed in all assays in random order. For in vitro experiments, same reagents were used for all relevant assays without bias.
Blinding	The Investigators were not blinded to allocation during experiments since reported outcomes are based on objective measurements. Appropriate controls were included for every study design.

Reporting for specific materials, systems and methods

We require information from authors about some types of materials, experimental systems and methods used in many studies. Here, indicate whether each material, system or method listed is relevant to your study. If you are not sure if a list item applies to your research, read the appropriate section before selecting a response.

Materials & experimental systems

n/a	Involved in the study	
☒	☐	Antibodies
☒	☐	Eukaryotic cell lines
☒	☐	Palaeontology and archaeology
☐	☒	Animals and other organisms
☐	☒	Human research participants
☒	☐	Clinical data
☒	☐	Dual use research of concern

Methods

n/a	Involved in the study	
☒	☐	ChIP-seq
☒	☐	Flow cytometry
☒	☐	MRI-based neuroimaging

Animals and other organisms

Policy information about studies involving animals; ARRIVE guidelines recommended for reporting animal research

Laboratory animals	Male Sprague-Dawley rats, male, 7 to 8 weeks (230-260 g); SPF pigs, male and female, 5 to 6 months old, 40-70 kg.
Wild animals	The study did not involve wild animals
Field-collected samples	The study did not involve field-collected samples
Ethics oversight	National University of Singapore (NUS) Institutional Animal Care and Use Committee (R16-008 and R15-0165).

Note that full information on the approval of the study protocol must also be provided in the manuscript.

Human research participants

Policy information about studies involving human research participants

Population characteristics	healthy human volunteers, male, 21-80 years old with no history of any medical conditions
Recruitment	Recruitment by word of mouth; since objective analysis of blood coagulation parameters were performed and participants were healthy, there should be minimum self-selection bias
Ethics oversight	National University of Singapore Institutional Review Board (B-15-094)

Note that full information on the approval of the study protocol must also be provided in the manuscript.
